# A case of metastatic leptomeningeal carcinomatosis from early gastric carcinoma

**DOI:** 10.1186/1477-7819-10-74

**Published:** 2012-05-03

**Authors:** Kwang-Kuk Park, Song-I Yang, Kyung-Won Seo, Young-Ok Kim, Ki-Young Yoon

**Affiliations:** 1 Department of Surgery, Kosin University College of Medicine, Busan, South Korea; 2 Department of Pathology, Kosin University College of Medicine, Busan, South Korea

**Keywords:** Early gastric cancer, Carcinomatosis, Leptomeningeal, Signet ring cell

## Abstract

Metastatic leptomeningeal carcinomatosis is estimated to occur in 3% to 8% of solid carcinomas. The most common causes of leptomeningeal carcinomatosis are breast cancer, lung cancer and malignant melanoma. Leptomeningeal carcinomatosis associated with gastric cancer, especially in its early stages, is exceedingly rare. Its presenting symptoms include headache, nauseaand seizures. In this report, we describe a case of leptomeningeal metastasis that presented with early-stage gastric cancer. A 67-year-old woman with a history of early-stage gastric cancer in remission was admitted to our hospital with 3 days of headache and nausea. Her gastric cancer had been treated 29 months prior to presentation by a radical subtotal gastrectomy with a Billroth I anastomosis. She had an uneventful recovery until she was diagnosed with metastases to the left axilla and neck 26 months after surgery. Her presenting symptoms of headache and nausea prompted cytologic examination of the cerebrospinal fluid and measurement of tumor markers, which revealed metastatic leptomeningeal carcinomatosis from her gastric cancer. This report aims to raise awareness of the possibility that even early-stage gastric cancer can lead to leptomeningeal carcinomatosis.

## Background

Gastric cancer is one of the most commonly diagnosed malignancies in Korea, and the third most common cause of mortality [1]. Most patients with gastric cancer eventually develop cachexia and peritoneal carcinomatosis and die of multiple organ failure. Early gastric cancer is defined as adenocarcinoma of the stomach confined to the mucosa or submucosa, regardless of the presence of lymph node metastasis [2]. Although the 5-year survival rate of early gastric cancer exceeds 85% in most series [3], some cases are associated with distant metastasis. Brain metastasis of gastric cancer in particular is rare, and leptomeningeal carcinomatosis is even less common. Leptomeningeal carcinomatosis is defined as the dissemination and growth of cancer cells within the leptomeningeal space. Metastatic leptomeningeal carcinomatosis is estimated to occur in 3% to 8% of solid carcinomas [4]. In this report, we present a rare case of early gastric cancer leading to leptomeningeal carcinomatosis.

## Case presentation

A 69-year-old woman with a history of early gastric cancer was admitted to our hospital with a 3-day history of general weakness, headache and nausea. On physical examination she was pale and appeared acutely ill, but was oriented to person, place and time. Her vital signs were as follows: blood pressure 130/75 mmHg, pulse rate 83 beats/min and respiratory rate 20 breaths/min. She exhibited no focal neurological signs, including abnormal reflexes, sensory deficits, nystagmus or neck stiffness. Her conjunctivae were pale and sclerae were anicteric. Breath sounds were clear and heart sounds were regular and without murmur.

**Figure 1:**
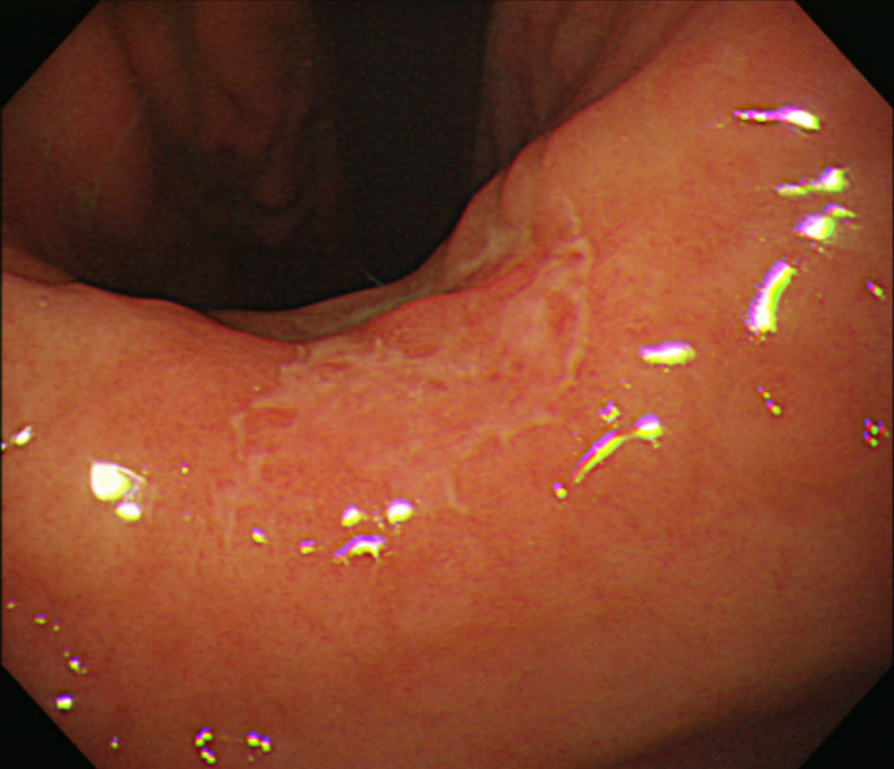
The gastroscopy showed a slightly depressed legion on gastric angle and posterior wall of the stomach.

The patient had been diagnosed with early gastric cancer 29 months prior to presentation, and had undergone a radical subtotal gastrectomy with Billroth I anastomosis and D1+ lymphadenectomy (Figure 1). Histology performed at the time had shown diffuse-type gastric adenocarcinoma confined to the mucosa with the presence of signet ring cells. No metastases were found in the 34 dissected lymph nodes. Her postoperative course was uneventful. She visited the surgery outpatient clinic for follow-up 6 weeks, 3 months and 6 months after surgery, and at 6-month intervals thereafter. She also underwent gastric endoscopy and abdominal computed tomography (CT) every 6 months. There was no evidence of recurrence until 26 months after surgery, when the patient complained of palpable masses in her left neck and axilla. She subsequently underwent neck CT, fluorine-18-fluorodeoxyglucose positron emission tomography, and fine needle aspiration of the left supraclavicular lymph node. Neck CT showed enlargement of multiple nodes in the left neck and axilla, including the left supraclavicular lymph node, and fine needle aspirate histology showed metastatic adenocarcinoma. She was diagnosed with recurrence of early gastric cancer and scheduled to receive systemic chemotherapy with leucovorin, fluorouracil and oxaliplatin (FOLFOX-6). She received 100 mg/m^2^ oxaliplatin and 100 mg/m^2^ leucovorin on the first day of treatment, followed by 2,400 mg/m^2^ of 5-fluorouracil on the first and second days of treatment every 2 weeks. After four cycles of chemotherapy, tumor response evaluation showed that disease stability had been achieved, prompting a fifth cycle of the same chemotherapy regimen.

**Figure 2:**
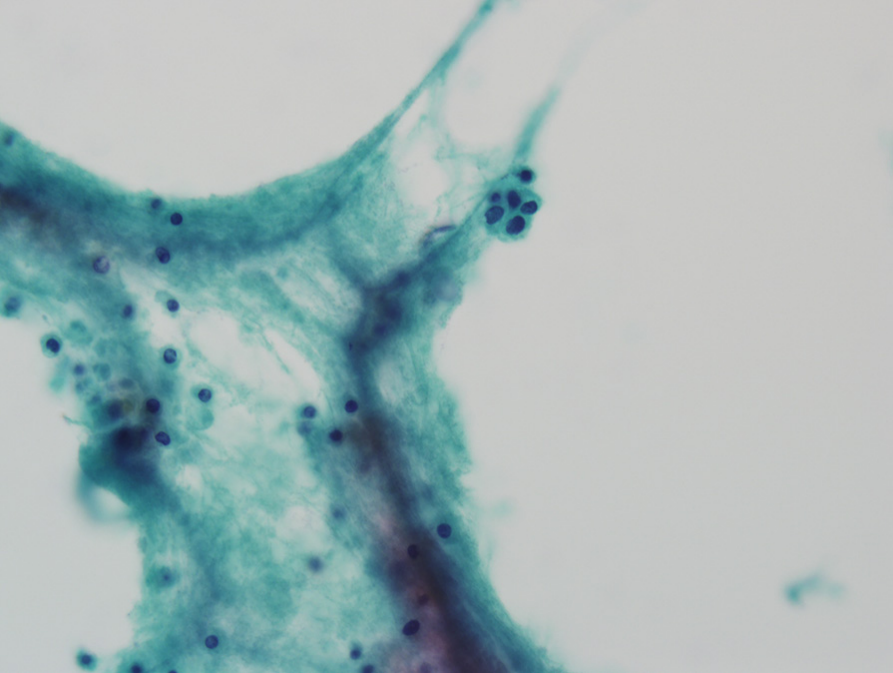
**Cytopathologic finding of cerebrospinal fluid reveals a small nest in scattered lymphocytic background.** This nest consisted of atypical round epithelial cells with large hyperchromatic nuclei (Papanicolaou stain, ×400).

She presented as an outpatient 7 days after completing the fifth chemotherapy cycle, complaining of general weakness, headache and nausea. A lumbar puncture was performed, and analysis of the cerebrospinal fluid (CSF) showed malignant cells (Figure 2), elevated protein, normal glucose, and the presence of tumor markers carcinoembryonic antigen and carbohydrate antigen19-9 She was diagnosed with metastatic leptomeningeal carcinomatosis of early gastric cancer and admitted to the hospital. On the seventh day of admission, she complained of dyspnea and her level of consciousness decreased, prompting intubation and admission to the intensive care unit. Symptomatic treatment was initiated with steroids and mannitol. Unfortunately, the patient died on the 20^th^ day of admission.

## Discussion

Early gastric cancer is defined as adenocarcinoma confined to the mucosa or submucosa (T1), irrespective of lymph node metastasis [2]. Its excellent 5-year survival rate, above 85% in most series, is attributable to the low incidence of extragastric extension of the disease. Lymph node metastasis is found in 1.9% to 7% of patients with mucosal and 7.4% to 20.9% of patients with submucosal invasion, and is associated with a worse prognosis [5,6]. The lymphatic and hematogenous extension of early gastric cancer can be explained by the infiltration of lymphatic and vascular drainage routes by malignant cells in the mucosal and submucosal layers. The prognostic significance of signet ring cells is controversial, with some authors reporting a common association with lymph node metastasis [7], and others reporting a better prognosis in the presence of signet ring cells [8].

First reported in the 1870 s, leptomeningeal carcinomatosis is most often caused by breast cancer, lung cancer and malignant melanoma [9]. Gastric cancer rarely causes leptomeningeal carcinomatosis; one study reported an incidence of 0.06% of all gastric cancers [10]. The clinical manifestations of leptomeningeal carcinomatosis are neurologic in origin and therefore variable, such as headache, nausea, vomiting and ataxia. The median time to diagnosis of leptomeningeal carcinomatosis from the initial cancer diagnosis had been reported between 76 days [11] and 17 months [10].

A diagnosis of leptomeningeal carcinomatosis is based on cytologic examination of the CSF in addition to imaging studies such as gadolinium-enhanced magnetic resonance imaging [12]. CSF findings include increased opening pressure, pleocytosis, elevated protein concentration, decreased glucose concentration and the presence of tumor markers, such as carcinoembryonic antigen and carbohydrate antigen19-9 in the case of gastric cancer. A definitive diagnosis of leptomeningeal carcinomatosis can be established only by documenting the presence of malignant cells in the CSF. Although Wasserstrom et al. reported that the first CSF sampling has a relatively low diagnostic sensitivity of 54%, with repeated sampling this ratio increases up to 91% [13]. In our patient, the first CSF cytology revealed malignant cells. It has been reported that imaging studies are abnormal in 67% of patients with leptomeningeal carcinomatosis [4].

Although leptomeningeal carcinomatosis is extremely rare in early gastric cancer [4], in South Korea it is most often associated with poorly differentiated adenocarcinoma with signet ring cell features [3]. Once leptomeningeal carcinomatosis develops from any primary cancer, prognosis is poor and neurological symptoms reduce quality of life. There is no standard treatment established for leptomeningeal carcinomatosis, although intrathecal chemotherapy is preferred as most intravenous chemotherapy agents do not penetrate the blood–brain barrier. Intraventricular chemotherapy with or without radiation is most commonly used in treatment. Intrathecal chemotherapy most often comprises methotrexate, cytarabine, thiotepa and steroids. The prolongation of survival afforded by intrathecal chemotherapy in leptomeningeal carcinomatosis is a matter of debate. Unfortunately, the prognosis of leptomeningeal carcinomatosis complicating gastric cancer is worse than in other solid cancers, with an overall median survival ranging from 4 to 7 weeks. [3,4]. However, a recent paper published in 2011 reported a more favorable outcome with triple combination therapy comprising intrathecal chemotherapy, whole brain irradiation and ventriculoperitoneal shunt compared with combination therapy (intrathecal chemotherapy plus whole brain irradiation) and monotherapy (intrathecal chemotherapy alone) [14].

## Conclusion

In conclusion, leptomeningeal carcinomatosis associated with gastric carcinoma is rare, especially in early stages of gastric cancer, and carries a poor prognosis. New neurological symptoms in patients with gastric cancer, including early-stage, should alert physicians to evaluate for nervous system involvement and promptly establish diagnostic and therapeutic plans.

## Consent

Written consent was obtained from the patient for the use and publication of this case report and the accompanying images. A copy of the written consent is available for review from the Editor-in-Chief of this journal.

## Ethical approval

This manuscript was approved by Kosin University College of Medicine I.R.B (Institutional Review Board).

## Competing interests

The authors declare that they have no competing interests.

## Authors’ contributions

PKK collected the information, researched the literature, and wrote the article, YSI and SKW helped with literature research and in preparing the manuscript, KYY performed the histological examination and helped prepare the manuscript, YKY helped in literature research and edited the final version of manuscript. All authors read and approved the final manuscript.
